# Carbapenemase-Producing Elizabethkingia Meningoseptica from Healthy Pigs Associated with Colistin Use in Spain

**DOI:** 10.3390/antibiotics8030146

**Published:** 2019-09-11

**Authors:** Pedro Miguela-Villoldo, Marta Hernández, Miguel Á. Moreno, David Rodríguez-Lázaro, Alberto Quesada, Lucas Domínguez, María Ugarte-Ruiz

**Affiliations:** 1 VISAVET Health Surveillance Centre, Universidad Complutense, Madrid 28040, Spain; 2 Departamento de Sanidad Animal, Facultad de Veterinaria, Universidad Complutense, 28040 Madrid, Spain; 3 Laboratorio de Biología Molecular y Microbiología, Instituto Tecnológico Agrario de Castilla y León, 47071 Valladolid, Spain; 4 Área de Microbiología, Departamento de Biotecnología y Ciencia de los Alimentos, Universidad de Burgos, 09001 Burgos, Spain; 5 Departamento de Bioquímica, Biología Molecular y Genética, Facultad de Veterinaria, Universidad de Extremadura, 10003 Cáceres, Spain; 6 INBIO G+C, Universidad de Extremadura, 10003 Cáceres, Spain

**Keywords:** carbapenemase, colistin, *Elizabethkingia meningoseptica*, Antimicrobial resistance

## Abstract

Carbapenems are considered last-resort antimicrobials, especially for treating infections involving multidrug-resistant Gram-negative bacteria. In recent years, extended-spectrum β-lactamase (ESBL) and carbapenemase-producing Gram-negative bacteria have become widespread in hospitals, community settings, and the environment, reducing the range of effective therapeutic alternatives. The use of colistin to treat infection caused by these multi-drug bacteria may favour the selection and persistence of carbapenem-resistant bacteria. In this study, it is described, for the first time to our knowledge, a carbapenemase-producing isolate of *Elizabethkingia meningoseptica* from healthy pigs in Spain. The isolate we report was recovered during a study to detect colistin-resistant bacteria from faecal samples of healthy food-production animals using a chromogenic selective medium. Unexpectedly, we found an isolate of *Elizabethkingia meningoseptica* with high Minimum Inhibitory Concentration (MIC) values for several antibiotics tested. Molecular analysis did not show any *mcr* family genes related with colistin resistance, but two carbapenemase genes, *bla*_B-12_1_ and *bla*_GOB-17_1_, were detected. This finding in healthy animals could suggest that colistin may favour the selection and persistence of carbapenem-resistant bacteria.

Carbapenems are considered last-resort antimicrobials, especially for treating infections involving multidrug-resistant Gram-negative bacteria [1]. In recent years, extended-spectrum β-lactamase (ESBL) and carbapenemase-producing Gram-negative bacteria have become widespread in hospitals, community settings and the environment, reducing the range of effective therapeutic alternatives [1]. The most common carbapenemases include metallo-β-lactamases (MBLs), *Klebsiella pneumoniae* carbapenemases (KPC) and class D oxacillinases [1]. Since the first detection in 2011 in Germany, various European countries have reported carbapenemase-producing *Escherichia coli* and *Salmonella* in food-producing animals [2]. In Spain, although described in pets and synanthropic animals [3,4], carbapenemase-producing *Escherichia coli* has not been detected in food-producing animals. To our knowledge, this is the first report of a carbapenemase-producing isolate of *Elizabethkingia meningoseptica* from healthy pigs in Spain.

*Elizabethkingia* species (genus formerly known as *Chryseobacterium*) are Gram-negative bacilli commonly found in freshwater, saltwater, soil and hospital environments [5]. Infections caused by *Elizabethkingia* spp., particularly *E. meningoseptica*, have been reported and are associated with a high fatality risk, particularly in immunocompromised patients [5].

The isolate we report was recovered during a study to detect colistin-resistant bacteria [6]. Antimicrobial chemotherapy, particularly colistin, has been the treatment of first choice to control Gram-negative bacteria in pig production, habitually used as a prophylactic [7]. Currently, the use of colistin is only allowed to treat infections caused by multidrug-resistant bacteria and metaphylaxis [8]. To carry out this study, we used a chromogenic selective medium. Identification involved mass spectrometry using a Bruker Daltonics UltrafleXtrem MALDI TOF/TOF instrument (Bruker Daltonics, Bremen, Germany) and Whole Genome Sequencing (WGS) to confirm the species and to further characterize the isolate. *Elisabethkingia meningoseptica* isolate was, 99% identity (accession number: NZ_CP016378.1).

Phenotypic characterization of antimicrobial susceptibility was performed. Minimal inhibitory concentrations (MICs) were determined using a two-fold broth microdilution reference method, according to ISO 20776-1:2006 [9]. Due to broad resistance of the *Elisabethkingia* genus and the diverse therapeutic alternatives described in literature for infections associated with this bacterium, two different antimicrobial panels (EUVSEC and EUST) were used, covering different antimicrobial families commonly used for Gram-negative and Gram-positive bacteria (Trek Diagnostic Systems, US). The isolate showed a carbapenemase-producing profile: high MIC values for carbapenems (meropenem MIC = 16 mg/L; imipenem MIC = 16 mg/L; ertapenem MIC > 2 mg/L). This isolate also presented high MIC values for colistin (MIC > 16 mg/L), ampicillin (MIC > 64 mg/L), gentamicin (MIC = 32 mg/L), ceftazidime (MIC = 128 mg/L), and cefotaxime (MIC = 32 mg/L). These results agree with reports that *Elizabethkingia* species are resistant to polymyxins, tetracycline and, especially, β-lactams, including carbapenems, due to the production of chromosomal MBLs. Two types of MBLs have been identified in *E. meningoseptica* (BlaB and GOB), and both types of MBLs can be found in the same strain. BlaB-12 and GOB-17 have been reported in a clinical human isolate in Korea [5]. We tested antimicrobials usually employed to treat Gram-positive infections, notably vancomycin and rifampicin which have been used to treat human infections caused by *E. meningoseptica* [5]. Our isolate had a vancomycin MIC of 8 µg/mg and rifampicin MIC of 0.25 µg/mg.

We determined the whole genome sequence of the isolate. Sequencing libraries were prepared using the Nextera XT kit and sequenced on a MiSeq (Illumina, San Diego, CA, USA) using v3 reagents with 2 × 300 cycles. The resistome of the draft genome was searched against ResFinder database [10] revealing two carbapenemase-encoding genes, *bla*_B-12_1_ (98% identity) and *bla*_GOB-17_1_ (100% identity). No other antimicrobial resistance genes were detected. The putative presence of plasmids was evaluated by BLASTn searches against the Plasmid Finder database [11]. No plasmids were found, indicating that the carbapenemase genes *bla*_B-12_1_ and *bla*_GOB-17_1_ were on the chromosome, consistent with what is commonly found for MBLs in this species [5]. Further analysis to confirm this finding are underway.

The presence of ESBL- and carbapenemase-producing *E. coli* in livestock has been associated with colistin resistance [1,12,13,14] and we observed in a previous study that Colistin-resistant *E. coli* in pigs has also been linked to colistin sales [6]. Colistin has been the treatment of first choice to control these bacteria in pig production and it has habitually used as a prophylactic approach as well [7]. Over the past decade, resistance to colistin has emerged becoming a public health problem mainly due to the spread of plasmid mobile genes in the *mcr* family. Thus, these colistin resistance genes could co-occur with ESBL and/or carbapenemase genes in the same isolate, promoting the co-selection. Because of that fact, these data suggest that colistin use may favour the selection and persistence of carbapenem-resistant bacteria. Further studies are needed to assess whether indeed colistin use in food animals, especially in pigs, is associated with a risk of co-selection of colistin, and more surprisingly, carbapenem resistance.
